# Exploring brain-glioma interaction: Effect of neuroligin-3 expression on neurocognitive functioning is independent from epilepsy

**DOI:** 10.1093/noajnl/vdag123

**Published:** 2026-05-11

**Authors:** Eva A Krijnen, Emma van Kessel, Angelika Mühlebner, Sven van Kempen, Wim Van Hecke, Martine J E van Zandvoort, Pierre A Robe, Tom J Snijders

**Affiliations:** Neurology and Neurosurgery, UMC Utrecht Brain Center, University Medical Center Utrecht, Utrecht, The Netherlands; Neurology and Neurosurgery, UMC Utrecht Brain Center, University Medical Center Utrecht, Utrecht, The Netherlands; Department of Pathology, University Medical Center Utrecht, Utrecht, The Netherlands; Department of Pathology, University Medical Center Utrecht, Utrecht, The Netherlands; Department of Pathology, University Medical Center Utrecht, Utrecht, The Netherlands; Neurology and Neurosurgery, UMC Utrecht Brain Center, University Medical Center Utrecht, Utrecht, The Netherlands; Neurology and Neurosurgery, UMC Utrecht Brain Center, University Medical Center Utrecht, Utrecht, The Netherlands; Neurology and Neurosurgery, UMC Utrecht Brain Center, University Medical Center Utrecht, Utrecht, The Netherlands

**Keywords:** cognition, diffuse glioma, epilepsy, neuroligin-3

## Abstract

**Background:**

Neurocognitive deficits are common in patients with diffuse glioma, compromising quality of life and prognosis. Neuroligin-3 is a postsynaptic activity-dependent protein, considered a key component of brain-glioma interactions, specifically at neurogliomal synapses. Neuroligin-3 may modulate neuronal and epileptic activity, contributing to both tumor growth and cognitive deficits. We aimed to investigate the relationship between intratumoral neuroligin-3 expression and neurocognitive functioning (NCF) in diffuse glioma patients, and to determine whether epilepsy modifies this relationship.

**Methods:**

In this single-center retrospective study, adult patients with grades 2-4 diffuse glioma underwent neuropsychological assessment prior to awake surgery. Tissue microarray analysis was used to assess intratumoral neuroligin-3 expression. We examined the relation between NCF (attention and executive functioning, memory, language, visuospatial functioning and psychomotor speed) and neuroligin-3 expression, using multivariable logistic regression models adjusting for tumor grade, location and volume. Additionally, presence of epilepsy and its interaction term with neuroligin-3 expression were added to separate models.

**Results:**

In total, 101 patients were included for analyses. Neuroligin-3 showed a protective effect on memory performance in all patients, not modified by epilepsy. Patients with neuroligin-3-positive high-grade and isocitrate dehydrogenase (IDH)-wild type glioma have a decreased risk of memory and attention and executive functioning deficits, again not modified by epilepsy. In low-grade and IDH-mutant glioma patients, neuroligin-3 expression was not associated with NCF.

**Conclusions:**

Intratumoral neuroligin-3 expression is an independent determinant of NCF, especially in the domain memory and in high-grade and IDH-wild type glioma. This protective effect was not modified by presence of epilepsy.

## Graphical Abstract

**Graphical Abstract:**
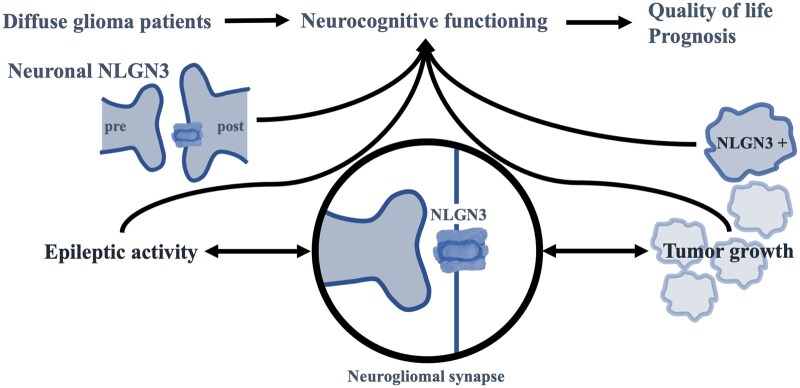

Key Points

- Neuroligin-3 expression relates to neurocognition in glioma patients.
- This effect is most obvious for IDH-wild type gliomas and the memory domain.
- In clinical trials involving neuroligin-3-targeting, cognition should be monitored.

Importance of the Study

Neuroligin-3 has been implicated in glioma growth through mechanisms involving synaptic signaling and patient survival. This study adds a new dimension by showing that neuroligin-3 is also associated with cognitive function in patients with diffuse glioma. These findings align with emerging concepts of glioma integration into functional brain networks, suggesting that tumor-related neuronal activity affects not only oncological outcomes but also cognition. Understanding how neuroligin-3-related signaling contributes to both tumor progression and cognitive impairment provides a critical link between molecular and network-level processes. As neuroligin-3 signaling may become a pharmacological target, these results underscore the need to monitor cognitive outcomes in future therapeutic studies.

Diffuse gliomas are progressive brain tumors with a generally poor prognosis and limited treatment options. In addition, ∼60% of patients with treatment-naïve diffuse glioma experiences neurocognitive deficits in 1 or multiple cognitive domains, including executive and visuospatial functioning, attention, memory, language, and psychomotor speed. These deficits are more common in patients with high-grade glioma (HGG) than with low-grade glioma (LGG).^1^ Neurocognitive dysfunction strongly limits quality of life and is an independent predictor of survival.^2^ Although literature regarding neurocognitive functioning (NCF) in patients with diffuse glioma is growing, the exact pathophysiology underlying these deficits is not yet fully understood.^1^

## Neuroligin-3, Epilepsy, and Tumor Growth

Diffuse gliomas have widespread effects on local and global functional networks, resulting in reduced global efficiency and disturbances in functional connectivity.^3-7^ Brain network disturbances are correlated with poor NCF and presence of epileptic seizures.^3^^,^^8-11^ Previous work demonstrated that direct (bidirectional) synaptic communication between neurons and glioma cells exists, driving tumor progression as well as neuronal (hyper)excitability.^7^^,^^12-16^ Neuronal activity, including epileptic activity, can generate synchronized postsynaptic currents in glioma networks via these neurogliomal synapses, promoting tumor progression.^12^ Neuroligin-3 is a postsynaptic cell-adhesion protein that plays a critical signaling role in this neuron-to-glioma communication, that, once released, promotes tumor growth.^17^ Neuroligin-3 is secreted in response to neuronal activity and induces its own expression in tumor cells.^13^^,^^18^ Expression of neuroligin-3 in tumor cells is positively correlated with levels of peritumoral and global oscillatory brain activity.^19^ Moreover, neuroligin-3 promotes cell metabolism, survival and proliferation via inducing the activity of a specific intracellular signaling pathway.^13^^,^^20^ Neuroligin-3 expression in tumor cells is strongly negatively correlated with patients’ overall survival.^13^^,^^14^ Hence, previous work suggested targeting neuroligin-3 secretion to slow tumor progression and improve overall survival,^14^ although potential adverse effects of modulating this pathway still need to be clarified.

## Neuroligin-3 and Cognition

In the adult brain, NCF relies on neuron-to-neuron and neuron-to-glia synaptic interactions that shape both local circuits and broader cognitive networks.^17^ In physiologic situations, neuroligin-3 is essential for synaptic function. In contrast to other neuroligins, neuroligin-3 is present in both excitatory and inhibitory synapses.^21^ Neuroligins are the ligands for neurexins, forming a transsynaptic neurexin-neuroligin complex. The family of neurexins and neuroligins shape synaptic plasticity and efficacy, and have been associated with cognitive functioning in various conditions, such as autism spectrum disorder.^22^ These cognitive disorders might be caused by an imbalance of inhibitory and excitatory synaptic transmissions. A recent study found that a subpopulation of HGG tumor cells can promote synaptogenesis and remodeling of functional connectivity, negatively affecting cognitive performance.^7^ Neuroligin-3 might play a role in this remodeling of neuronal circuits, and may therefore lead to alterations in distinct cognitive processes.^22^^,^^23^

**Figure 1.**
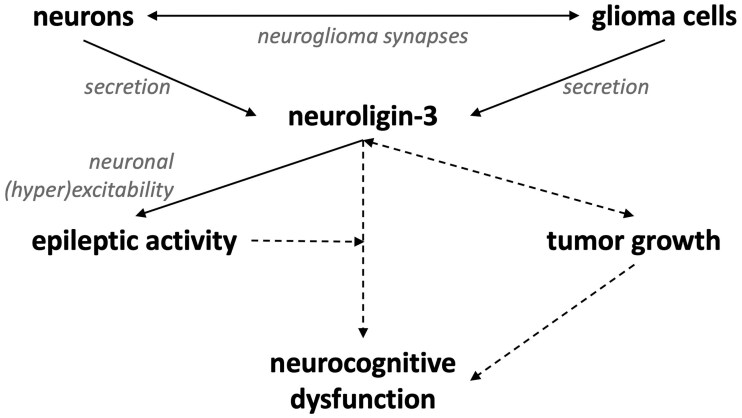
Schematic overview of the relationships between neuronal activity, neuroligin-3 secretion, tumor growth, epileptic activity, and neurocognitive dysfunction in diffuse glioma. Dashed lines denote the relationship explored in this study.

Further insight into the pathogenesis of neurocognitive dysfunction in patients with diffuse glioma could identify prognostic determinants and treatment targets, and is therefore highly sought after. Evidence supporting the close relation between tumor and brain activity, and between this altered brain activity and NCF is increasing. Since neuronal activity, including epileptic activity, is affected by neuroligin-3, the level of neuroligin-3 expression in the tumor might relate to NCF. The aim of present study is to assess the relation between intratumoral neuroligin-3 expression and NCF in patients with diffuse glioma, and whether this relation is affected by the presence of epilepsy (Figure 1). Further insights into this interrelationship can guide the development of new interventions to improve cognitive outcome, and possibly epilepsy burden and survival, in glioma patients.

## Methods

### Participants

A retrospective cohort study of 793 consecutive adult patients with proven diffuse glioma was conducted at the University Medical Center Utrecht. All patients were diagnosed with diffuse glioma according to the World Health Organization (WHO) 2016 classification of gliomas,^24^ and had first surgical tumor resection under either general anesthesia (“nonawake cohort,” *n *= 596) or awake conditions (“awake cohort,” *n *= 197) in the period of January 2010 to January 2017. The awake cohort was included in this study. The nonawake cohort served as control group to assess generalizability of our results of the awake cohort to the general population of diffuse glioma patients. Included patients underwent neuropsychological assessment prior to awake surgery, as part of routine clinical care. Exclusion criteria were (1) any antitumor therapy (surgery, radio- or chemotherapy) given before neuropsychological assessment, (2) incomplete neuropsychological assessment data, defined as data obtained on <4 domains or if <50% of the tasks per domain were completed, (3) unavailable or not assessable tissue for tissue microarray (TMA) analysis. Patients receiving anticonvulsants or corticosteroids at time of neuropsychological assessment were not excluded. Patient characteristics were retrospectively retrieved from the electronic patient files. Those included age, sex, WHO 2016 classification of gliomas, Karnofsky Performance Status,^25^ epilepsy at presentation, and tumor volume and localization. Three-dimensional volumes were measured with the use of Osirix Lite (version 9.5.2) on T2-/fluid-attenuated inversion recovery-weighted MRI scans by experienced clinical scientists (E.A.K./E.v.K.) under the supervision of a neuro-oncological neurosurgeon (P.A.R.) and neurologist (T.J.S.). The volume was defined as the whole area of hyperintensity, representing total lesion volume, including tumor infiltration and edema. The collection of neuropsychological assessment and TMA data are described below.

The current study includes a targeted analysis of data acquired for a prior study investigating tumor-related molecular determinants of neurocognitive deficits. This study was approved by the medical ethics committee of the University Medical Center Utrecht (19-101).^26^

### Neuropsychological Tests

The neuropsychological instruments used for neuropsychological assessment prior to awake surgery are listed in Table 1. These tests are standardized psychometric instruments to assess functioning of the major neurocognitive domains.^27–40^ All test scores were corrected for the normal effects of age and, if applicable, educational level. Classification of neuropsychological tests into cognitive domains often varies in the literature, and most neuropsychological tests highlight more than 1 cognitive domain. We applied the predetermined test classification in our analysis, as previously published (Table 1).^26^ Within each domain, patients were considered impaired if they performed below −1.0 SD on any of the administered tests highlighting that specific domain. To distinguish between severe, moderate, and mild neurocognitive dysfunction, we applied 3 thresholds to report deviations: −2.0 SD, −1.5 SD, and −1.0 SD.

**Table 1.** Neuropsychological tests for neuropsychological assessment per cognitive domain

Attention and executive functioning
Wechsler Adult Intelligence Scale III digit span forward^27^ ^,^ ^28^
Trail making test switching ratio^29^
Phonologic fluency^30^ ^,^ ^31^
Stroop/Delis Kaplan executive function system inhibition ratio^32^ ^,^ ^33^
Language
Boston naming test^34^
Token test^35^
Memory
Wechsler Adult Intelligence Scale III digit span backward^27^ ^,^ ^28^
Rey auditory verbal learning test immediate, delay and recognition (Dutch Version)^36^ ^,^ ^37^
Rey-Osterieth complex figure test delay^38^ ^,^ ^39^
Semantic fluency^31^
Psychomotor speed
Stroop/Delis Kaplan executive function system I^32^ ^,^ ^33^
Stroop/Delis Kaplan executive function system II^32^ ^,^ ^33^
Trail making test-A^29^
Visuospatial functioning
Judgment of line orientation^40^
Rey-Osterieth complex figure test delay^38^ ^,^ ^39^

### Immunohistochemistry

Expression of neuroligin-3 in tumor cells was examined with the use of TMA techniques. Construction of TMA blocks and immunohistochemical staining were completed at the department of Pathology at the University Medical Center Utrecht. Formalin-fixed, paraffin-embedded tumor tissues of all 793 patients with diffuse glioma were derived from the archive of neurosurgical resections between 2010 and 2017. Tumor tissues from both awake and nonawake surgeries were obtained. For core sampling, 1 board-certified neuropathologist (W.V.H.) defined representative areas of tumor tissue. Three core samples were collected from glioma tissue of each patient. All samples were placed on recipient arrayed paraffin blocks. Immunohistochemical staining was performed on 4 µm sectioned TMA slides from the paraffin blocks. After deparaffinizing with xylene and rehydrating with graded ethanol solution, TMA slides were placed in citrate antigen-repairing solution (pH 6) for 12 min at 126°C. Peroxidase-blocking solutions (3% H_2_O_2_ solution) were added to block endogenous peroxidase. Tissue microarray slides were then incubated with antineuroligin-3 (Novus NBP1-90080, 1:25 dilution).

To assess in which cell types neuroligin-3 is expressed, we performed double immunohistochemical staining, on 4 µm sectioned slides from the formalin-fixed paraffin-embedded blocks. Slides were deparaffinized in xylene, rehydrated with graded ethanol solution and rinsed in deionized water, after which slides were placed in a peroxidase blocking solution. Antigen retrieval was performed in an ethylenediaminetetraacetic acid (EDTA) buffer (pH 9) at 100°C for 20 min. The slides were incubated for 60 min with use of the following primary antibodies: antiglial fibrillary acidic protein (GFAP; Roche EP672Y Rabbit, RTU dilution), anti-NeuN (Milllipore A60 Mouse 1:1500 dilution), antimicrotubule-associated protein 2 (MAP2; Sigma HM-2 Mouse 1:4000), anti-isocitrate dehydrogenase (IDH) (Bio SB IHC132 1:200), and antineurofilament (CellMarque 2F11 Mouse RTU dilution). To prevent cross-linking from antibodies, the rabbit slides were boiled for 10 min in EDTA buffer (pH 9) before incubating with the neuroligin-3 antibody. After a quality check for the first staining, neuroligin-3 was incubated overnight at 4°C. Slides were counterstained for 30 s using 1:4 diluted Mayer’s hematoxylin and rinsed in tap water for 10 min. Slides were dried and cover slipped using the Thermo Scientific Clearvue.

### Evaluation of Immunohistochemistry

A medical student (E.A.K.) analyzed all immunostained tissue sections, supervised by a board-certified neuropathologist (A.M.). Tumor cells were considered neuroligin-3 positive if the entire membrane or cytoplasm was stained, in line with the information provided by the protein atlas.^41^ Expression of neuroligin-3 was evaluated semiquantitative based on percentage of positive tumor cells per 100 tumor cells. Since TMA analysis with the same antineuroligin-3 has not yet been used for assessing neuroligin-3 expression in diffuse gliomas, we could not compare our methods with previous literature. A prior study investigating neuroligin-3 expression by means of TMA analysis with another antineuroligin-3 classified core samples into low, moderate, or high staining, without specifying exact cutoff values.^19^ In the current study, the expression levels of the 3 core samples of each patient were averaged. Subsequently, dichotomization of mean expression of core samples was performed based on data-driven median split of neuroligin-3 expression in the awake cohort. Due to the minimal expression observed in the awake cohort, a 3-level classification was not feasible, and dichotomization was therefore chosen to ensure a meaningful and robust analysis.

To assess in which cell type neuroligin-3 is expressed, additional staining was performed in a small subset of grade 2/3 astrocytoma IDH-mutant (*n *= 3) and IDH-wild type (*n *= 2) tumor, grade 2/3 oligodendroglioma IDH-mutant tumor (*n *= 3) and grade 4 glioblastoma (*n *= 4) samples with the use of GFAP for astrocytes and glial cells, NeuN and neurofilament for neuronal cell bodies and dendrites, CD68 for tumor-associated macrophages, and IDH and MAP2 for tumor cells.

### Statistical Analysis

Statistical analysis was performed with the use of IBM SPSS statistics 25.0. for Macintosh (SPSS Inc.). Deficits in any domain and all 5 domains separately were chosen as outcome measures. Analyses were performed for the 3 thresholds specified above (≤−1.0 SD, ≤−1.5 SD, and ≤−2.0 SD). Baseline characteristics were reported as frequencies with valid percentages and medians with interquartile range. *P*≤.05 were considered statistically significant.

To test the external validity of neuroligin-3 expression to the general population of patients with diffuse glioma, both mean and dichotomous neuroligin-3 expression were compared between the awake and nonawake cohort by means of an independent-sample *t*-test and chi-square test, respectively.

Univariable binary logistic regression analyses were performed to evaluate the association between neuroligin-3 expression and all cognitive outcome measures. Based on the literature and univariable binary logistic regression for NCF, tumor grade, volume and location, and epilepsy at presentation were identified as possible confounders in the relationship between neuroligin-3 and NCF.^1^^,^^42–46^ Potential multicollinearity between confounders was detected with use of formal detection-tolerances and variance inflation factors of confounders, and Pearson’s correlations coefficients between confounders. Confounders with a tolerance of <0.10, variance inflation factor >10, or a correlation coefficient of <−0.40 or >0.40 were considered as multicollinear. We detected possible outliers with Mahalanobis distance.

Initial multivariable binary logistic regression analyses for all outcome measures only corrected for tumor grade, since associations between tumor volume/location and cognitive outcomes are established, but their relationship with neuroligin-3 expression is not yet clear. Therefore, tumor volume and location were only included in models showing (near) significant results, defined as *P*<.10, to avoid overadjustment.

To test whether epilepsy at presentation is an effect modifier in the relationship between neuroligin-3 on NCF, we first applied multivariable analyses, correcting for tumor grade, volume and location, and epilepsy at presentation, followed by the inclusion of an interaction term between epilepsy and neuroligin-3. Based on the level of significance (*P* ≤.05) of the included covariates, that is, the presence of epilepsy or its interaction term with neuroligin-3, covariates were retained in the model.

Finally, we performed predefined subgroup analyses for LGG patients (grade 2/3 astrocytoma IDH-mutant and oligodendroglioma IDH-mutant 1p/19q-codeletion) and HGG patients (grade 2/3 astrocytoma IDH-wild type and grade 4 glioblastoma IDH-wild type, and grade 4 astrocytoma, IDH-mutant), as well as for IDH-wild type and -mutant patients, separately. Subgroup analyses included the same steps as analyses of the total awake cohort.

## Results

### Clinical Characteristics

A total of 101 patients, who underwent awake surgery between 2010 and 2017 and of whom TMA data were assessable, were included. Descriptive characteristics are presented in Table 2. Age ranged from 21 to 81 years old, and tumor volume from 2.80 to 277.78 cm^3^. An HGG was diagnosed in 51.1% of patients. In 65.3%, the tumor was located in the left hemisphere.

**Table 2.** Patient characteristics of included participants

Patient characteristics	Included participants (*N* = 101)
Sex (female)	33 (32.7%)
Age at first surgery	54 (37-65)
Karnofsky performance scale	
<70	8 (7.9%)
≥70	91 (91.9%)
Tumor volume (cm^3^)	58.57 (27.04-95.73)
WHO 2016 classification	
Grade 2/3 astrocytoma IDH-M	24 (23.8%)
Grade 2/3 oligodendroglioma IDH-M 1p19q	22 (21.8%)
Grade 2/3 astrocytoma IDH-WT	8 (7.9%)
Grade 4 glioblastoma IDH-M	2 (2.0%)
Grade 4 glioblastoma IDH-WT	45 (44.6%)
Location	
Left hemisphere	66 (65.3%)
Right hemisphere	34 (33.7%)
Both hemispheres	1 (1.0%)
Presence of epilepsy (at presentation)	63 (64.9%)

Demographics are shown as frequency with valid percentage or median with interquartile range (IQR).

Abbreviations: IDH, isocitrate dehydrogenase; M, mutant; WHO, World Health Organization; WT, wild type.

Neuropsychological assessment data were missing in 5.9% and 7.9% of patients for domains visuospatial functioning and language, respectively. Other domains had missing values in 2.0%-3.0% of patients.

### Neuroligin-3 Expression

Median expression of neuroligin-3 was 0% positive cells with a range from 0% to 20%. Therefore, we dichotomized all mean scores into score 0, corresponding to 0% positive tumor cells, and score 1, corresponding to ≥1% positive tumor cells. Of 101 patients, 54 patients (53.5%) had negative neuroligin-3 expression (score 0). Forty-seven patients had at least 1% neuroligin-3-positive tumor cells (score 1).

Expression was determined in both the awake and nonawake cohorts. Expression of neuroligin-3 was assessable in 448 patients of the nonawake cohort. Mean neuroligin-3 expression was not significantly different between the awake and nonawake cohorts (*t*(547)=0.267, *P *= .790). After dichotomization, neuroligin-3 expression was significantly higher in the awake cohort compared to the nonawake cohort (odds ratio [OR], 95% CI=1.800, 1.162-2.790, *P *= .008).

Double staining of antiglial fibrillary acidic protein (GFAP; A, C, and E), IDH (B), CD68 (D), and antineuroligin-3 in low-grade (A-B) and high-grade (C) IDH-mutant tumor tissue and the core (D) and border zone (E) of low-grade IDT-wild type tumor tissue. Arrows highlight the expression of GFAP in astrocytes (A, C, and E), IDH-positive tumor cells (B) and CD68^+^ in tumor-associated macrophages (D).

**Figure 2.**
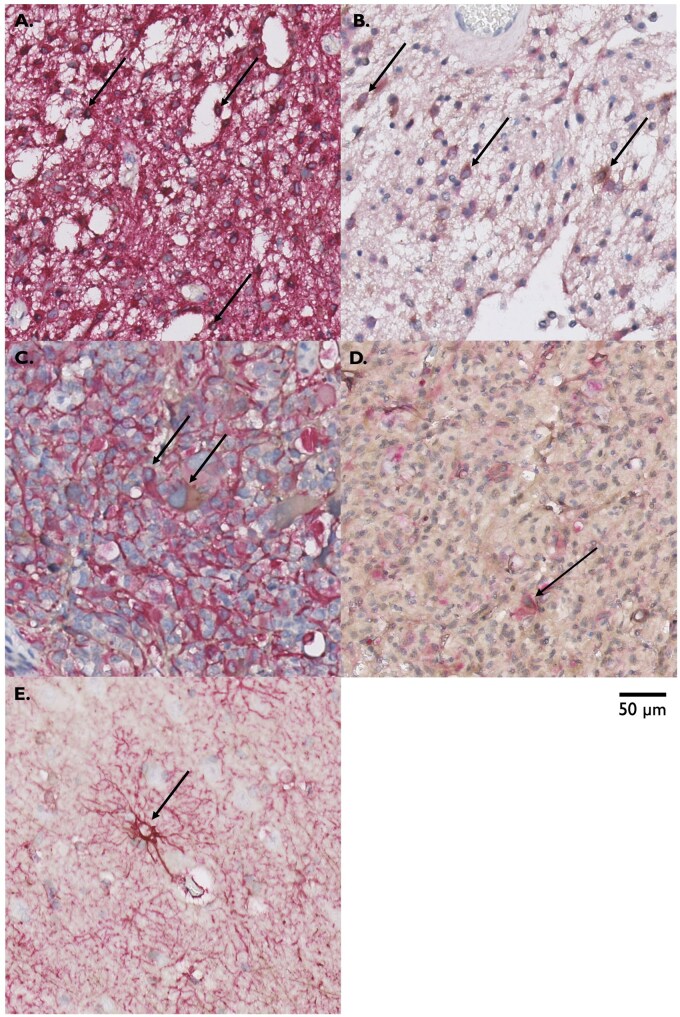
Differences between low-grade glioma and high-grade glioma in the expression of neuroligin-3 in reactive astrocytes. Double staining of antiglial fibrillary acidic protein (GFAP; A, C, and E), isocitrate dehydrogenase (IDH; B), CD68 (D) and antineuroligin-3 in low-grade (A, B) and high-grade (C) IDH-mutant tumor tissue and the core (D) and border zone (E) of low-grade IDH-wild type tumor tissue. Arrows highlight the expression of GFAP in astrocytes (A, C, and E), IDH-positive tumor cells (B), and CD68^+^ in tumor-associated macrophages (D).

In LGG, we identified a colocalization pattern of neuroligin-3 and GFAP as well as IDH in the tumor core (Figure 2A and B). In the border zone of the tumor, coexpression of neuroligin-3 and GFAP was also present, which confirmed the presence of neuroligin-3 expression in both reactive astrocytes and tumor cells (Figure 2E). In HGG, neuroligin-3 did not colocalize with GFAP-positive cells in the tumor core, which is suggestive for expression of neuroligin-3 only in tumor cells (Figure 2C). Only minimal overlap was observed between CD68^+^ and neuroligin-3, indicating very limited coexpression of neuroligin-3 in tumor-associated macrophages in LGG (Figure 2D), whereas no overlap was detected in HGG. There was no coexpression of neuroligin-3 in either NeuN-positive cells, that is, neuronal cell bodies, or neurofilament.

### Protective Effect of Neuroligin-3 on Cognition

Severe neurocognitive deficits (≤−2.0 SD) were found in 51.5% of patients in any domain, and in 15.1%-28.3% in all domains separately. Complete neuropsychological assessment data are displayed in Table S1.

Neuroligin-3 was univariably associated with a decreased risk of cognitive deficits (Table 3). Neuroligin-3 expression was significantly and negatively associated with deficits in any domain at ≤−2.0 SD. For the domain memory, we found significant results across all thresholds. The domain language showed significant results at both ≤−1.5 and ≤−2.0 SD. For attention and executive functioning, neuroligin-3 expression was only significantly associated with mild deficits (≤−1.0 SD).

**Table 3.** Univariable analyses of neuroligin-3 for neurocognitive deficits at various thresholds

Domain (≤−1.0 SD)	OR (95% CI)	*P-*value
Any domain	0.731 (0.257-2.082)	.557
Attention and executive functioning	0.396 (0.174-0.903)	.028^a^
Language	0.500 (0.209-1.196)	.119
Memory	0.397 (0.175-0.899)	.027^a^
Psychomotor speed	0.460 (0.198-1.067)	.070
Visuospatial functioning	0.568 (0.245-1.317)	.187

Domain (≤−1.5 SD)		
Any domain	0.671 (0.295-1.528)	.342
Attention and executive functioning	0.514 (0.228-1.156)	.107
Language	0.351 (0.135-0.914)	.032^a^
Memory	0.288 (0.119-0.698)	.006^a^
Psychomotor speed	0.525 (0.212-1.296)	.162
Visuospatial functioning	0.729 (0.296-1.799)	.493

Domain (≤−2.0 SD)		
Any domain	0.390 (0.173-0.877)	.023^a^
Attention and executive functioning	0.435 (0.174-1.092)	.076
Language	0.253 (0.065-0.976)	.046^a^
Memory	0.258 (0.087-0.770)	.015^a^
Psychomotor speed	0.547 (0.214-1.396)	.207
Visuospatial functioning	1.037 (0.362-2.967)	.946

Results of univariable analyses of neuroligin-3 for deficits in any domain and all domains separately are reported as odds ratio (OR) with 95% confidence interval (CI) and corresponding *P*-value. Logistic regression models were assessed at 3 thresholds for neurocognitive deficits: at ≤−1.0 standard deviation (SD), ≤−1.5 SD and ≤−2.0 SD.

a Significant *P*-values.

Multivariable analyses correcting for tumor grade (WHO 2016 classification) showed a trend toward a protective effect of neuroligin-3 only on the domain memory at ≤−2.0 SD (OR, 95% CI=0.356, 0.106-1.193, *P *= .094; Table S2). Multivariable analyses adjusting for tumor grade, location and volume showed a significant decreased risk of severe memory deficits (≤−2.0 SD) in neuroligin-3-positive tumors (OR, 95% CI=0.243, 0.062-0.946, *P *= .031; Table 4 Model 1).

**Table 4.** Multivariable analyses of neuroligin-3 for memory deficits

Determinant	OR (95% CI)	*P-*value
Model 1		
Neuroligin-3	0.243 (0.062-0.946)	.031^a^
WHO 2016 (grade 2/3 astrocytoma IDH-M=reference)		.958
Grade 2/3 oligodendroglioma IDH-M 1p19q	0.000 (0.000-∞)	.998
Grade 2/3 astrocytoma IDH-WT	1.041 (0.074-14.618)	.999
Grade 4 glioblastoma IDH-M	4.165 (0.069-2.515×10^2^)	.582
Grade 4 glioblastoma IDH-WT	2.733 (0.469-15.922)	.455
Tumor volume (cm^3^)	1.000 (1.000-1.000)	.059
Location (left hemisphere=reference)		.982
Right hemisphere	1.265 (0.337-4.751)	.850
Bilateral hemispheres	2.524 (0.000-∞)	.000
Model 2		
Neuroligin-3	0.037 (0.200-0.995)	.037^a^
WHO 2016 (grade 2/3 astrocytoma IDH-M = reference)		.966
Grade 2/3 oligodendroglioma IDH-M 1p19q	0.000 (0.000-∞)	.998
Grade 2/3 astrocytoma IDH-WT	0.997 (0.064-15.526)	.998
Grade 4 glioblastoma IDH-M	2.319 (0.035-1.534×10^2^)	.694
Grade 4 glioblastoma IDH-WT	1.846 (0.280-12.170)	.524
Tumor volume (cm^3^)	1.000 (1.000-1.000)	.140
Location (left hemisphere=reference)		.999
Right hemisphere	1.035 (0.241-4.448)	.963
Bilateral hemispheres	3.228 (0.000-∞)	.000
Presence of epilepsy (at presentation)	0.546 (0.144-2.068)	.373
Model 3		
Neuroligin-3	0.271 (0.037-1.995)	.200
WHO 2016 (grade 2/3 astrocytoma IDH-M=reference)		.968
Grade 2/3 oligodendroglioma IDH-M 1p19q	0.000 (0.000-∞)	.998
Grade 2/3 astrocytoma IDH-WT	0.941 (0.058-15.290)	.966
Grade 4 glioblastoma IDH-M	2.109 (0.037-1.189×10^2^)	.717
Grade 4 glioblastoma IDH-WT	1.798 (0.268-12.073)	.546
Tumor volume (cm^3^)	1.000 (1.000-1.000)	.151
Location (left hemisphere=reference)		.000
Right hemisphere	1.007 (0.231-4.382)	.993
Bilateral hemispheres	4.443 (0.000-∞)	.000
Presence of epilepsy (at presentation)	0.628 (0.145-2.729)	.535
Presence of epilepsy×neuroligin-3	0.518 (0.026-10.170)	.665

Results of multivariable analysis of neuroligin-3 for memory deficits at ≤−2.0 standard deviation are reported as odds ratio (OR) with 95% confidence interval (CI) and corresponding *P*-value. In model 1, the multivariable logistic regression models were corrected for tumor grade, tumor volume and location on T_2_-weighted fluid-attenuated inversion recovery scans. In model 2, the multivariable logistic regression models were corrected for tumor grade, tumor volume and location on T_2_-weighted fluid-attenuated inversion recovery scans, and the presence of epilepsy at presentation. In model 3, the multivariable logistic regression models were corrected for tumor grade, tumor volume and location on T_2_-weighted fluid-attenuated inversion recovery scans, and epilepsy at presentation, including an interaction term with neuroligin-3 (epilepsy×neuroligin-3).

Abbreviations: IDH, isocitrate dehydrogenase; M, mutant; WHO, World Health Organization; WT, wild type.

a Significant *P*-values.

### Interaction between Epilepsy and Neuroligin-3 on Cognition

For the domain memory (≤−2.0 SD), we tested a multivariable regression model correcting for tumor grade, location and volume, as well as epilepsy at presentation, including an interaction term between epilepsy and neuroligin-3. The significant protective effect of neuroligin-3 on the domain memory retained after the inclusion of the presence of epilepsy as additional covariate (OR, 95% CI=0.037, 0.200-0.995), *P *= .037; Table 4 Model 2), but was diminished after inclusion of its interaction term with neuroligin-3 (Table 4 Model 3). Neither the interaction term nor the presence of epilepsy itself was associated with memory deficits. Hence, the protective effect of neuroligin-3 on cognition was not modified by epilepsy.

### Subgroup Analysis by Tumor Grade (WHO 2016)

Patient characteristics and neuropsychological assessment data of both LGG and HGG patients are listed in Tables S1 and S3, respectively. Tumors were neuroligin-3-positive in 63.0% of LGG patients and 32.7% of HGG patients. Severe deficits in any domain (≤−2.0 SD) were present in 22.7% of LGG patients and 74.5% of HGG patients.

Both univariable and multivariable (adjusting for tumor grade) analyses in the LGG subgroup showed no significant associations between neuroligin-3 and cognitive deficits (Table S4).

In contrast, in the HGG subgroup, neuroligin-3 expression was univariably significantly associated with memory functioning (≤−2.0 SD; OR, 95% CI=0.235, 0.058-0.952, *P *= .043) and attentional and executive functioning (≤−1.0 SD; OR, 95% CI=0.195, 0.052-0.734, *P *= .016; Table S5). Again, neuroligin-3 had a protective effect on NCF. After correcting for tumor grade, the domains memory (≤−2.0 SD; OR, 95% CI=0.223, 0.053-0.941, *P *= .041) and attention and executive functioning (≤−1.0 SD; OR, 95% CI=0.199, 0.052-0.764, *P *= .019) still showed a significant protective effect of neuroligin-3 expression on NCF. The multivariable analyses in HGG, in which we adjusted for tumor grade, location and volume, again showed a significant decreased risk of severe memory deficits (≤−2.0 SD; OR, 95% CI=0.091, 0.014-0.603, *P *= .013; Table S6) and mild deficits in attention and executive functioning (≤−1.0 SD; OR, 95% CI=0.200, 0.049-0.814, *P *= .025; Table S7) in neuroligin-3-positive tumors. After the inclusion of epilepsy at presentation as additional covariate, the significant effect of neuroligin-3 on severe memory deficits remained significant (OR, 95% CI=0.060, 0.006-0.614, *P *= .018; Table S8), and the effect on mild deficits in attention and executive functioning weakened (OR, 95% CI=4.271, 0.899-20.325, *P *= .068; Table S9). The multivariable analysis including the interaction term of epilepsy and neuroligin-3 showed a trend toward an effect of neuroligin-3 on severe memory deficits (OR, 95% CI=0.112, 0.008-1.499, *P *= .098; Table S10). Neither the interaction term nor the presence of epilepsy itself was associated with memory deficits. Hence, the protective effect of neuroligin-3 was not modified by epilepsy. Since this study was explorative, and no significant interaction between epilepsy and neuroligin-3 was found, epilepsy and its interaction term were excluded from the logistic regression model.

### Subgroup Analysis by IDH Status

We then assessed the effect of neuroligin-3 on NCF for IDH-wild type and -mutant subgroups. Patient characteristics and neuropsychological assessment data of both IDH-wild type and IDH-mutant patients are listed in Tables S1 and S3, respectively. Tumors were neuroligin-3-positive in 17% of IDH-wild type patients and 62.5% of IDH-mutant patients. Severe deficits in any domain (≤−2.0 SD) were present in 92.5% of IDH-wild type patients and 71.7% of IDH-mutant patients.

Both univariable and multivariable (adjusting for tumor grade) analyses in the IDH-mutant subgroup showed no significant associations between neuroligin-3 and cognitive deficits (Table S11). For the IDH-wild type subgroup, neuroligin-3 expression was univariably associated with memory functioning (≤−2.0 SD; OR, 95% CI=0.268, 0.065-1.097, *P *= .067) and attentional and executive functioning (≤−1.0 SD; OR, 95% CI=0.195, 0.052-0.734, *P *= .016; Table S12). Again, neuroligin-3 had a protective effect on NCF. Effects were independent of tumor grade for both domains (memory at ≤−2.0 SD; OR, 95% CI=0.267, 0.064-1.116, *P *= .070, attention and executive functioning at ≤−1.0 SD; OR, 95% CI=0.231, 0.059-0.907, *P *= .036).

The multivariable analyses for IDH-wild type tumors, adjusting for tumor grade, location and volume, again showed a significantly decreased risk of severe memory deficits (≤−2.0 SD; OR, 95% CI=0.105, 0.016-0.704, *P *= .020; Table S13) and of mild deficits in attention and executive functioning (≤−1.0 SD; OR, 95% CI=0.231, 0.055-0.965), *P *= .045; Table S14) in patients with neuroligin-3-positive tumors. After the inclusion of epilepsy at presentation as additional covariate, only the significant effect of neuroligin-3 on severe memory deficits remained significant (OR, 95% CI=0.072, 0.007-0.729), *P *= .026; Table S15), and the effect on mild deficits in attention and executive functioning diminished (OR, 95% CI=3.790, 0.775-18.540, *P *= .100; Table S16). Adding the interaction term of epilepsy and neuroligin-3 to the model yielded no significant effects of neuroligin-3, neither of the interaction term nor the presence of epilepsy on severe memory deficits (Table S17). Hence, the protective effect of neuroligin-3 was not modified by epilepsy.

## Discussion

In this study, we examined the relation between tumoral expression of neuroligin-3 and NCF in patients with diffuse glioma. After adjusting for tumor grade, location and volume, we found a significant protective effect of neuroligin-3 on the domain memory. This protective effect was not affected by the presence of epilepsy. In subgroup analyses of patients with HGG, we found a significantly decreased risk of deficits in domains memory and attention and executive functioning in neuroligin-3-positive tumors as well. Neuroligin-3 was not significantly associated with cognitive impairments in patients with LGG. In subgroup analyses according to IDH status, we only found protective effects of neuroligin-3-positivity for NCF in patients with IDH-wild type tumors.

Neuroligin-3 seemed to have a protective effect on cognition. The central nervous system preserves normal cognitive functioning through complex neuron-to-neuron and neuron-to-glia interactions to collectively establish cognitive brain networks. Neuroligin-3 has structural and functional effects on both excitatory and inhibitory synapses.^21^ It is essential for stabilization of neuronal networks and maintenance of proper synaptic transmission.^47^ While synaptic and cellular mechanisms maintain healthy network dynamics, these same processes may influence glioma behavior.^17^ This could be the reason neuroligin-3 is a key component for neurogliomal synapses, which bridge neuronal and tumor activity, ultimately promoting tumor growth.^12^ Hence, concurrently with the pro-oncogenic aspects of neuroligin-3, expression of neuroligin-3 might provide a more efficient functional brain network of communicating cells, necessary for NCF.^9^ It would be interesting to look in more detail at the relationship between neuroligin-3 and NCF in terms of functional connectivity, for example by resting-state functional network analysis with the use of functional magnetic resonance imaging^48^ or magnetoencephalography.^9^ Another possible explanation for the reduced risk of cognitive impairments in patients with neuroligin-3-positive diffuse glioma may be derived from the existence of a neuroligin-3 gain-of-function mutation, that has been associated with autism spectrum disorder.^49^ Regional altered synaptic function seemed to cause impaired social interaction but enhanced spatial learning abilities in patients with autism, accompanied by increased inhibitory synaptic transmission without affecting excitatory transmission.^50^ These findings indicate interfering with neuroligin-3 function alters the excitatory/inhibitory balance in synapses.^50^^,^^51^ Neuroligin-3 may therefore be an essential regulator of specific synaptic processes underlying cognitive functioning, especially memory functioning.^22^

The significant effects of neuroligin-3 specifically on memory functioning may reflect its broader role in protecting cognition as a whole. Memory is a multifaceted cognitive domain encompassing various subprocesses,^52^ making it a sensitive proxy for overall neurocognitive integrity. Therefore, the protective association observed in memory performance likely indicates more generalized cognitive preservation linked to neuroligin-3 expression. This interpretation aligns with the notion that neuroligin-3 regulates synaptic mechanisms fundamental to multiple cognitive domains, with memory providing the most measurable manifestation in this study. Future investigations should explore these broader cognitive effects through comprehensive assessments and advanced neuroimaging approaches.

Results of the double stainings in the small sample of different tumor tissues suggest neuroligin-3 is expressed in both reactive astrocytes and tumor cells in LGG, but only in tumor cells in HGG. Diffuse gliomas are known to induce reactive changes in the surrounding microenvironment, including the activation of astrocytes.^53^ In LGG, the relatively slow growth rate and less invasive behavior of the tumor may allow for a closer interaction between tumor cells and astrocytes, modulating microglia/astrocytes behavior. Therefore, the slow growth process observed in LGG may cause a higher level of interaction between tumor cells and preexisting astrocytes, which could explain the expression of neuroligin-3 in reactive astrocytes along with the expression in tumor cells. Another explanation for the differential expression pattern between HGG and LGG could be attributed to the genetic and molecular alterations, affecting the interaction between tumor cells and the surrounding tumor microenvironment, including astrocytes.^54^ Further research is needed to elucidate the precise mechanisms underlying the altered expression of neuroligin-3 in gliomas of different grades and to understand its functional implications in tumor development and progression.

Even after correcting for tumor grade and volume—both well-established predictors of poor NCF^1^^,^^55^—we observed a significant protective effect of neuroligin-3 on the domain memory. Previous studies investigating the functional aspects of neuroligin-3 in patients with diffuse glioma showed that tumoral neuroligin-3 was associated with synchronous neuronal and intratumoral electrical activity, promoting tumor growth.^13^^,^^19^ Interestingly, the fact that the association between neuroligin-3 expression and better NCF remained significant after correcting for tumor-related factors suggests that additional pathophysiological mechanisms are involved beyond tumor metabolism and growth alone. The reduced risk of cognitive deficits was especially clear in patients with faster growing and more aggressive tumors, who generally experience more cognitive deficits compared to patients with less aggressive tumors.^1^ This only emphasizes that the protective effect is independent of tumor growth and grade. The fact that no significant associations were found in LGG patients might be due to insufficient statistical power given the minor occurrence of cognitive deficits in LGG patients.

Our results showed no significant increase nor decrease of the risk of cognitive deficits in patients with both epilepsy and a neuroligin-3-positive tumor. Previous work showed that the severity of epilepsy and the use of antiepileptic drugs aggravate cognitive deficits in patients with LGG.^46^ Since neuroligin-3 expression depends on neuronal activity,^13^ epileptic activity was considered as possible factor interacting with neuroligin-3 expression in the context of NCF. Since epilepsy appears to catalyze tumor growth via neurogliomal synapses of which neuroligin-3 is an important factor,^12^ it would be worth investigating the etiological relationship between epilepsy and neuroligin-3 more thoroughly. Also, relevant in vitro and in vivo models are important for further mechanistic studies of neurogliomal synapses, associated brain network functioning and the possible role of epilepsy.

Our study has some limitations. We only included patients who underwent awake surgery. A previous study of our cohort showed that patients undergoing awake surgery are often selected based on localization of the tumor and have better clinical performance than patient who had surgery under general anesthesia.^56^ This selection of patients probably led to an underestimation of cognitive deficits in the complete population of patients with diffuse glioma. Also, we found a significant difference in dichotomous neuroligin-3 expression between the awake and nonawake cohorts. The finding that neuroligin-3 expression is higher in patients undergoing awake surgery, who have generally better clinical performance and therefore have probably less cognitive deficits, underscores the protective effect of neuroligin-3 on cognition. However, both the selection of patients and the difference in expression levels might reduce the generalizability of our results. Our results are also subject to common limitations of TMA analysis.^57^ Single-observer assessment of core samples as well as sampling error due to heterogeneity in gliomas could have caused less accurate TMA data. Accuracy might have also been affected by missing core samples and the choice of antibody. If one or more core samples per patients were missing, mean expression score was based on less samples of the tumor. Staining of the antibody we used for immunohistochemistry was low in intensity and quantity. Besides, we dichotomized the core sample mean expression of neuroligin-3 at the median expression level of 0.0% positive cells per 100 cells, meaning core sample means of only 1.0% positive cells were considered positive. This narrowed the distinction between negative and positive expression levels. However, the possible imprecision of the assessment increases the risk of false negative results, which makes our significant findings even more robust.

In conclusion, tumoral expression of neuroligin-3, an activity-dependent mitogen, is independently associated with better NCF in patients with diffuse glioma, particularly in those with HGG. This protective effect, most pronounced in the memory domain, seems not modified by epilepsy. These findings suggest that neuroligin-3 may play a beneficial role in preserving cognition, highlighting a complex dual function in glioma biology: promoting tumor growth while supporting cognitive networks. Given this potential tradeoff, we strongly recommend further investigation into the role of neuroligin-3 in cognitive outcomes before pursuing therapeutic strategies aimed at inhibiting its expression to slow tumor progression or reduce recurrence rate, as suggested by previous research.^14^ Targeting neuroligin-3 without understanding its cognitive impact could be detrimental. In clinical trials involving neuroligin-3-targeted drugs, NCF should be closely monitored.

## Supplementary Material

vdag123_Supplementary_Data

## Data Availability

The tabulated data that support the findings of this study are available from the corresponding author upon reasonable request from a qualified investigator.
