# Supplementary material for: Exploring brain-glioma interaction: Effect of neuroligin-3 expression on neurocognitive functioning is independent from epilepsy
Source: Neurooncol Adv. 2026 May 11;8(1):vdag123. doi: 10.1093/noajnl/vdag123 (PMC13259598; doi:10.1093/noajnl/vdag123)
Supplement: vdag123_Supplementary_Data [file vdag123_supplementary_data.docx]

**Supplementary table 1. Neuropsychological assessment data of all included participants**

| **Domain** | **Total** | **LGG** | **HGG** | **IDH-WT** | **IDH-M** |
| --- | --- | --- | --- | --- | --- |
| Any domain  ≤-1.0 SD  ≤-1.5 SD  ≤-2.0 SD | 82 (82.8)  63 (63.6)  51 (51.5) | 31 (70.5)  17 (38.6)  10 (22.7) | 51 (92.7)  46 (83.6)  41 (74.5) | 49 (92.5)  44 (83.0)  40 (75.5) | 33 (71.7)  19 (41.3)  11 (23.9) |
| Attention and executive functioning  ≤-1.0 SD  ≤-1.5 SD  ≤-2.0 SD | 59 (59.6)  43 (43.4)  28 (28.3) | 10 (22.7)  7 (15.9)  7 (15.9) | 42 (76.4)  33 (60.0)  21 (38.2) | 41 (77.4)  33 (62.3)  21 (39.6) | 18 (39.7)  10 (21.7)  7 (15.2) |
| Language  ≤-1.0 SD  ≤-1.5 SD  ≤-2.0 SD | 33 (35.5)  27 (29.0)  14 (15.1) | 6 (14.0)  4 (9.3)  2 (4.7) | 27 (54.0)  23 (46.0)  12 (24.0) | 33 (71.7)  19 (41.3)  11 (23.9) | 7 (15.9)  5 (11.4)  3 (6.8) |
| Memory  ≤-1.0 SD  ≤-1.5 SD  ≤-2.0 SD | 57 (57.6)  36 (36.4)  22 (22.2) | 14 (31.8)  6 (13.6)  2 (4.5) | 43 (78.2)  30 (54.5)  20 (36.4) | 41 (77.4)  29 (54.7)  19 (35.8) | 16 (34.8)  7 (15.2)  3 (6.5) |
| Psychomotor speed  ≤-1.0 SD  ≤-1.5 SD  ≤-2.0 SD | 37 (37.8)  28 (28.6)  25 (25.5) | 10 (22.7)  5 (11.4)  4 (9.1) | 27 (50.0)  23 (42.6)  21 (38.9) | 26 (50.0)  23 (44.2)  21 (40.4) | 11 (23.9)  5 (10.9)  4 (8.7) |
| Visuospatial functioning  ≤-1.0 SD  ≤-1.5 SD  ≤-2.0 SD | 37 (38.9)  27 (28.4)  17 (17.9) | 6 (13.6)  4 (9.1)  1 (2.3) | 31 (60.8)  23 (45.1)  16 (31.4) | 29 (59.2)  21 (42.9)  16 (32.7) | 8 (17.4)  6 (13.0)  1 (2.2) |

Neuropsychological assessment data of all included participants are reported as frequency with valid percentage. Values are shown for the total cohort, low-grade glioma cohort (LGG), high-grade glioma cohort (HGG), isocitrate dehydrogenase-wild type (IDH-WT) and IDH-mutant (IDH-M) patients. SD = standard deviation.

**Supplementary table 2. Multivariable analyses correcting for tumor grade in the total awake cohort**

| **Domain (≤-1.0 SD)** | **OR (95% CI)** | ***P*-value** |
| --- | --- | --- |
| Any domain  Attention and executive functioning  Language  Memory  Psychomotor speed  Visuospatial functioning | 1.606 (0.461 – 5.594)  0.613 (0.245 – 1.534)  0.920 (0.327 – 2.590)  0.696 (0.249 – 1.948)  0.676 (0.265 – 1.723)  1.072 (0.346 – 3.323) | 0.457  0.295  0.875  0.491  0.412  0.904 |
| **Domain (≤-1.5 SD)** | **OR (95% CI)** | ***P*-value** |
| Any domain  Attention and executive functioning  Language  Memory  Psychomotor speed  Visuospatial functioning | 1.549 (0.526 – 4.564)  0.888 (0.340 – 2.317)  0.569 (0.192 – 1.682)  0.436 (0.159 – 1.197)  0.888 (0.316 – 2.498)  1.320 (0.418 – 4.170) | 0.427  0.808  0.308  0.107  0.823  0.636 |
| **Domain (≤-2.0 SD)** | **OR (95% CI)** | ***P*-value** |
| Any domain  Attention and executive functioning  Language  Memory  Psychomotor speed  Visuospatial functioning | 0.632 (0.223 – 1.788)  0.571 (0.205 – 1.594)  0.388 (0.091 – 1.645)  0.356 (0.106 – 1.193)  1.002 (0.336 – 2.995)  2.869 (0.787 – 10.458) | 0.387  0.285  0.199  0.094**  0.997  0.110 |

Results of multivariable analyses of neuroligin-3 for deficits in any domain and all domains separately are reported as odds ratio (OR) with 95% confidence interval (CI) and corresponding *P*-value. Logistic regression models were corrected for WHO 2016 classification and assessed at three thresholds for neurocognitive deficits: at ≤-1.0 standard deviation (SD), ≤-1.5 SD and ≤-2.0 SD. Near significant results (*P*<0.10) are marked with double asterisks (**).

**Supplementary table 3. Patient characteristics of subgroups**

| **Patient characteristics** | **LGG** | **HGG** | **IDH-WT** | **IDH-M** |
| --- | --- | --- | --- | --- |
| **N** | 46 (100%) | 55 (100%) | 53 (100%) | 48 (100%) |
| **Sex** (female) | 18 (39.1%) | 15 (27.3%) | 14 (26.4%) | 19 (39.6%) |
| **Age at first surgery** | 42 [34.5 – 52] | 63 [54 – 67] | 64 [55.5 – 68] | 41 [31 – 51.75] |
| **Karnofsky Performance Scale**  <70  ≥70 | 1 (2.2%)  45 (97.8%) | 7 (12.7%)  48 (87.3%) | 6 (11.3%)  47 (88.7%) | 2 (4.2%)  46 (95.8%) |
| **Tumor volume** (cm^3^) | 45.9 [25.2 – 66.0] | 75.9 [37.2 – 151.3] | 75.9 [34.8 – 149.3] | 51.0 [26.0 – 67.9] |
| **WHO 2016 classification**  Grade 2/3 astrocytoma IDH-M  Grade 2/3 oligodendroglioma IDH-M 1p19q  Grade 2/3 astrocytoma IDH-WT  Grade 4 glioblastoma IDH-M  Grade 4 glioblastoma IDH-WT | 24 (52.2%)  22 (47.8%)  NA  NA  NA | NA  NA  8 (8.3%)  2 (4.2%)  45 (87.5%) | NA  NA  8 (8.3%)  NA  45 (87.5%) | 24 (50.0%)  22 (45.8%)  NA  2 (4.2%)  NA |
| **Location**  Left hemisphere  Right hemisphere  Both hemispheres | 26 (56.5%)  19 (41.3%)  1 (2.2%) | 40 (72.7%)  15 (27.3%)  0 (0.0%) | 38 (71.7%)  15 (28.3%)  0 (0.0%) | 28 (58.3%)  19 (39.6%)  1 (2.1%) |
| **Presence of epilepsy** (at presentation) | 30 (68.2%) | 33 (62.3%) | 33 (64.7%) | 30 (65.2%) |
| **Neuroligin-3 expression**  0.0%  ≥1.0% | 17 (37.0%)  29 (63.0%) | 37 (67.3%)  18 (32.7%) | 36 (67.9%)  17 (32.1%) | 18 (37.5%)  30 (62.5%) |

Patient characteristics of subgroups are reported for subgroups: low-grade glioma (LGG), high-grade glioma (HGG), isocitrate dehydrogenase-wild type (IDH-WT) and IDH-mutant (IDH-M) patients. Demographics are shown as frequency with valid percentage or median with interquartile range (IQR). WHO = World Health Organization; NA = not applicable.

**Supplementary table 4**. **Univariable and multivariable analyses correcting for tumor grade in low-grade glioma patients**

|  | **Univariable analyses** |  | **Multivariable analyses** | |
| --- | --- | --- | --- | --- |
| **Domain (≤-1.0 SD)** | **OR (95% CI)** | ***P*-value** | **OR (95% CI)** | ***P*-value** |
| Any domain  Attention and executive functioning  Language  Memory  Psychomotor speed  Visuospatial functioning | 1.136 (0.298 – 4.330)  1.650 (0.452 – 6.026)  0.480 (0.084 – 2.740)  0.667 (0.181 – 2.452)  0.478 (0.114 – 2.004)  1.167 (0.189 – 7.207) | 0.851  0.449  0.409  0.542  0.313  0.868 | 1.489 (0.357 – 6.211)  1.857 (0.484 – 7.127)  0.563 (0.094 – 3.363)  0.819 (0.210 – 3.196)  0.595 (0.134 – 2.645)  0.805 (0.117 – 5.542) | 0.585  0.367  0.529  0.774  0.495  0.826 |
| **Domain (≤-1.5 SD)** | **OR (95% CI)** | ***P*-value** | **OR (95% CI)** | ***P*-value** |
| Any domain  Attention and executive functioning  Language  Memory  Psychomotor speed  Visuospatial functioning | 1.650 (0.452 – 6.026)  1.444 (0.316 – 6.598)  0.500 (0.063 – 3.962)  0.520 (0.092 – 2.948)  0.840 (0.125 – 5.645)  1.800 (0.171 – 18.911) | 0.449  0.635  0.512  0.460  0.858  0.624 | 1.857 (0.484 – 7.127)  1.738 (0.358 – 8.435)  0.502 (0.060 – 4.161)  0.617 (0.103 – 3.682)  0.951 (0.134 – 6.746)  1.071 (0.089 – 12.831) | 0.367  0.493  0.523  0.596  0.960  0.957 |
| **Domain (≤-2.0 SD)** | **OR (95% CI)** | ***P*-value** | **OR (95% CI)** | ***P*-value** |
| Any domain  Attention and executive functioning  Language  Memory  Psychomotor speed  Visuospatial functioning | 1.444 (0.316 – 6.598)  0.722 (0.140 – 3.731)  0.519 (0.030 – 8.928)  1.242E8 (0.0E0 – ∞)  1.800 (0.171 – 18.911)  5.983E7 (0.0E0 – ∞) | 0.635  0.698  0.651  0.999  0.624  0.999 | 1.335 (0.281 – 6.337)  0.669 (0.123 – 3.638)  0.520 (0.028 – 9.539)  6.350E7 (0.0E0 – ∞)  2.506 (0.221– 28.377)  2.997E7 (0.0E0 – ∞) | 0.716  0.642  0.660  0.998  0.458  0.998 |

Results of univariable and multivariable analyses of neuroligin-3 for deficits in any domain and all domains in the low-grade glioma subgroup are reported separately as odds ratio (OR) with 95% confidence interval (CI) and corresponding *P*-value. Multivariable logistic regression models were corrected for tumor grade. All analyses were assessed at three thresholds for neurocognitive deficits: at ≤-1.0 standard deviation (SD), ≤-1.5 SD and ≤-2.0 SD.

**Supplementary table 5 Univariable and multivariable analyses correcting for tumor grade in high-grade glioma patients**

|  | **Univariable analyses** |  | **Multivariable analyses** |  |
| --- | --- | --- | --- | --- |
| **Domain (≤-1.0 SD)** | **OR (95% CI)** | ***P*-value** | **OR (95% CI)** | ***P*-value** |
| Any domain  Attention and executive functioning  Language  Memory  Psychomotor speed  Visuospatial functioning | 1.500 (0.145 – 15.523)  0.195 (0.052 – 0.734)  1.143 (0.346 – 3.777)  0.607 (0.162 – 2.269)  0.716 (0.230 – 2.231)  1.111 (0.329 – 3.753) | 1.000  0.016*  0.827  0.458  0.564  0.865 | 2.063 (0.146 – 29.180)  0.199 (0.052 – 0.764)  1.171 (0.331 – 4.145)  0.560 (0.116 – 2.694)  0.733 (0.221 - 2.432)  1.238 (0.308 – 4.980) | 0.592  0.019*  0.807  0.469  0.612  0.764 |
| **Domain (≤-1.5 SD)** | **OR (95% CI)** | ***P*-value** | **OR (95% CI)** | ***P*-value** |
| Any domain  Attention and executive functioning  Language  Memory  Psychomotor speed  Visuospatial functioning | 0.968 (0.212 – 4.411)  0.542 (0.173 – 1.701)  0.600 (0.178 – 2.022)  0.387 (0.122 – 1.232)  0.795 (0.251 – 2.521)  1.333 (0.407 – 4.369) | 0.966  0.294  0.410  0.108  0.795  0.635 | 1.104 (0.184 – 6.616)  0.568 (0.164 – 1.969)  0.595 (0.169 – 2.095)  0.370 (0.108 – 1.268)  0.865 (0.255 – 2.930)  1.393 (0.385 – 5.044) | 0.914  0.372  0.419  0.114  0.816  0.614 |
| **Domain (≤-2.0 SD)** | **OR (95% CI)** | ***P*-value** | **OR (95% CI)** | ***P*-value** |
| Any domain  Attention and executive functioning  Language  Memory  Psychomotor speed  Visuospatial functioning | 0.367 (0.105 – 1.286)  0.505 (0.149 – 1.709)  0.343 (0.066 – 1.794)  0.235 (0.058 – 0.952)  0.700 (0.214 – 2.285)  2.247 (0.647 – 7.804) | 0.117  0.272  0.205  0.043*  0.555  0.203 | 0.319 (0.074 – 1.382)  0.521 (0.142 – 1.912)  0.352 (0.065 – 1.915)  0.223 (0.053 – 0.941)  0.750 (0.209 – 2.691)  2.726 (0.729 – 10.193) | 0.127  0.326  0.227  0.041*  0.659  0.136 |

Results of univariable and multivariable analyses of neuroligin-3 for deficits in any domain and all domains in the high-grade glioma subgroup are reported separately as odds ratio (OR) with 95% confidence interval (CI) and corresponding *P*-value. Multivariable logistic regression models were corrected for tumor grade. All analyses were assessed at three thresholds for neurocognitive deficits: at ≤-1.0 standard deviation (SD), ≤-1.5 SD and ≤-2.0 SD. Significant *P*-values are marked with an asterisk(*).

**Supplementary table 6 Multivariable analyses correcting for tumor grade, volume and location in high-grade glioma patients – memory**

| Determinant | OR (95% CI) | *P*-value |
| --- | --- | --- |
| Neuroligin-3 | 0.091 (0.014 – 0.603) | 0.013* |
| WHO 2016 (Grade 2/3 astrocytoma IDH-WT = reference)  Grade 4 glioblastoma IDH-M  Grade 4 glioblastoma IDH-WT | 8.593 (0.048 – 1.552**⋅**10^3^)  3.468 (0.302 – 39.802) | 0.565  0.417  0.318 |
| Tumor volume (cm^3^) | 1.00 (1.00 – 1.00) | 0.094 |
| Location Right hemisphere (vs left) | 2.984 (0.584 – 15.253) | 0.189 |

Results of multivariable analysis of neuroligin-3 for memory deficits at ≤-2.0 standard deviation in the high-grade glioma subgroup are reported as odds ratio (OR) with 95% confidence interval (CI) and corresponding *P*-value. Multivariable logistic regression models were corrected for tumor grade, tumor volume and location on T_2_-weighted Fluid-Attenuated Inversion Recovery scans. Significant *P*-values are marked with an asterisk(*). WHO = World Health Organization; IDH = isocitrate dehydrogenase; WT = wild type; M = mutant.

**Supplementary table 7 Multivariable analyses correcting for tumor grade, volume and location in high-grade glioma patients – attention and executive functioning**

| Determinant | OR (95% CI) | *P*-value |
| --- | --- | --- |
| Neuroligin-3 | 0.200 (0.049 – 0.814) | 0.025* |
| WHO 2016 (Grade 2/3 astrocytoma IDH-WT = reference)  Grade 4 glioblastoma IDH-M  Grade 4 glioblastoma IDH-WT | 0.303 (0.005 – 18.022  1.437 (0.211 – 9.775) | 0.649  0.567  0.711 |
| Tumor volume (cm^3^) | 1.00 (1.00 – 1.00) | 0.380 |
| Location Right hemisphere (vs left) | 0.679 (0.149 – 3.103) | 0.618 |

Results of multivariable analysis of neuroligin-3 for attention and executive functioning deficits at ≤-1.0 standard deviation in the high-grade glioma subgroup are reported as odds ratio (OR) with 95% confidence interval (CI) and corresponding *P*-value. Multivariable logistic regression models were corrected for tumor grade, tumor volume and location on T_2_-weighted Fluid-Attenuated Inversion Recovery scans. Significant *P*-values are marked with an asterisk(*). WHO = World Health Organization; IDH = isocitrate dehydrogenase; WT = wild type; M = mutant.

**Supplementary table 8 Multivariable analyses correcting for tumor grade, volume and location, and presence of epilepsy in high-grade glioma patients – memory**

| Determinant | OR (95% CI) | *P*-value |
| --- | --- | --- |
| Neuroligin-3 | 0.060 (0.006 – 0.614) | 0.018* |
| WHO 2016 (Grade 2/3 astrocytoma IDH-WT = reference)  Grade 4 glioblastoma IDH-M  Grade 4 glioblastoma IDH-WT | 5.438 (0.022 – 1.327**⋅**10^3^)  2.406 (0.194 – 29.822) | 0.754  0.546  0.494 |
| Tumor volume (cm^3^) | 1.000 (1.000 – 1.000) | 0.176 |
| Location Right hemisphere (vs left) | 2.621 (0.455 – 15.112) | 0.281 |
| Presence of epilepsy (at presentation) | 0.507 (0.108 – 2.389) | 0.390 |

Results of multivariable analysis of neuroligin-3 for memory deficits at ≤-2.0 standard deviation in the high-grade glioma subgroup are reported as odds ratio (OR) with 95% confidence interval (CI) and corresponding *P*-value. Multivariable logistic regression models were corrected for tumor grade, tumor volume and location on T_2_-weighted Fluid-Attenuated Inversion Recovery scans, and the presence of epilepsy at presentation. Significant *P*-values are marked with an asterisk(*). WHO = World Health Organization; IDH = isocitrate dehydrogenase; WT = wild type; M = mutant.

**Supplementary table 9 Multivariable analyses correcting for tumor grade, volume and location, and presence of epilepsy in high-grade glioma patients – attention and executive functioning**

| Determinant | OR (95% CI) | *P*-value |
| --- | --- | --- |
| Neuroligin-3 | 4.271 (0.899 – 20.325) | 0.068** |
| WHO 2016 (Grade 2/3 astrocytoma IDH-WT = reference)  Grade 4 glioblastoma IDH-M  Grade 4 glioblastoma IDH-WT | 0.466 (0.062 – 3.482)  0.185 (0.002 – 17.484) | 0.567  0.457  0.467 |
| Tumor volume (cm^3^) | 1.000 (1.000 – 1.000) | 0.116 |
| Location Right hemisphere (vs left) | 0.778 (0.133 – 4.563) | 0.781 |
| Presence of epilepsy (at presentation) | 0.411 (0.053 – 3.164) | 0.393 |

Results of multivariable analysis of neuroligin-3 for attention and executive functioning deficits at ≤-1.0 standard deviation in the high-grade glioma subgroup are reported as odds ratio (OR) with 95% confidence interval (CI) and corresponding *P*-value. Multivariable logistic regression models were corrected for tumor grade, tumor volume and location on T_2_-weighted Fluid-Attenuated Inversion Recovery scans, and the presence of epilepsy at presentation. Near significant results (*P*<0.10) are marked with double asterisks (**). WHO = World Health Organization; IDH = isocitrate dehydrogenase; WT = wild type; M = mutant.

**Supplementary table 10 Multivariable analyses correcting for tumor grade, volume and location, and an interaction term between neuroligin-3 and presence of epilepsy in high-grade glioma patients – memory**

| Determinant | OR (95% CI) | *P*-value |
| --- | --- | --- |
| Neuroligin-3 | 0.112 (0.008 – 1.499) | 0.098** |
| WHO 2016 (Grade 2/3 astrocytoma IDH-WT = reference)  Grade 4 glioblastoma IDH-M  Grade 4 glioblastoma IDH-WT | 4.604 (0.031 – 6.926**⋅**10^2^)  2.50 (0.194 – 32.230) | 0.754  0.551  0.483 |
| Tumor volume (cm^3^) | 1.00 (1.00 – 1.00) | 0.194 |
| Location Right hemisphere (vs left) | 2.654 (0.454 – 15.523) | 0.279 |
| Presence of epilepsy (at presentation) | 0.594 (0.120 – 2.929) | 0.522 |
| Presence of epilepsy*Neuroligin-3 | 0.00 (0.00 – ∞) | 0.999 |

Results of multivariable analysis of neuroligin-3 for memory deficits at ≤-2.0 standard deviation in the high-grade glioma subgroup are reported as odds ratio (OR) with 95% confidence interval (CI) and corresponding *P*-value. Multivariable logistic regression models were corrected for tumor grade, tumor volume and location on T_2_-weighted Fluid-Attenuated Inversion Recovery scans, and epilepsy at presentation, including an interaction term with neuroligin-3 (Epilepsy*Neuroligin-3). Near significant results (*P*<0.10) are marked with double asterisks (**). WHO = World Health Organization; IDH = isocitrate dehydrogenase; WT = wild type; M = mutant.

**Supplementary table 11**. **Univariable and multivariable analyses correcting for tumor grade in IDH-mutant patients**

|  | **Univariable analyses** |  | **Multivariable analyses** | |
| --- | --- | --- | --- | --- |
| **Domain (≤-1.0 SD)** | **OR (95% CI)** | ***P*-value** | **OR (95% CI)** | ***P*-value** |
| Any domain  Attention and executive functioning  Language  Memory  Psychomotor speed  Visuospatial functioning | 1.094 (0.291 – 4.109)  1.294 (0.375 – 4.468)  0.360 (0.069 – 1.870)  0.643 (0.185 – 2.234)  0.382 (0.096 – 1.526)  0.972 (0.201 – 4.700) | 0.894  0.683  0.224  0.487  0.173  0.972 | 1.489 (0.357 – 6.211)  1.444 (0.400 – 5.212)  0.563 (0.094 – 3.363)  0.819 (0.210 – 3.196)  0.470 (0.111 – 1.990)  0.805 (0.117 – 5.542) | 0.585  0.575  0.529  0.774  0.306  0.826 |
| **Domain (≤-1.5 SD)** | **OR (95% CI)** | ***P*-value** | **OR (95% CI)** | ***P*-value** |
| Any domain  Attention and executive functioning  Language  Memory  Psychomotor speed  Visuospatial functioning | 1.490 (0.433 – 5.121)  1.485 (0.328 – 6.717)  0.333 (0.049 – 2.249)  0.375 (0.073 – 1.930)  0.865 (0.130 – 5.778)  1.200 (0.196 – 7.362) | 0.449  0.608  0.259  0.241  0.881  0.844 | 1.857 (0.484 – 7.127)  1.738 (0.358 – 8.435)  0.502 (0.060 – 4.161)  0.442 (0.080 – 2.451)  0.951 (0.134 – 6.746)  1.071 (0.089 – 12.831) | 0.367  0.493  0.523  0.350  0.960  0.957 |
| **Domain (≤-2.0 SD)** | **OR (95% CI)** | ***P*-value** | **OR (95% CI)** | ***P*-value** |
| Any domain  Attention and executive functioning  Language  Memory  Psychomotor speed  Visuospatial functioning | 1.034 (0.253 – 4.22)  0.747 (0.146 – 3.825)  0.259 (0.022 – 3.114)  1.185 (0.099 – 14.136)  1.846 (0.177 – 19.306)  5.770**⋅**10^7^ (0.000 – ∞) | 0.963  0.726  0.287  0.893  0.609  0.999 | 0.983 (0.227 – 4.253)  0.669 (0.123 – 3.638)  0.520 (0.028 – 9.539)  1.047 (0.060 – 18.226)  2.506 (0.221– 28.377)  2.792**⋅**10^7^ (0.000 – ∞) | 0.982  0.642  0.660  0.975  0.458  0.458 |

Results of univariable and multivariable analyses of neuroligin-3 for deficits in any domain and all domains in the isocitrate dehydrogenase-mutant subgroup are reported separately as odds ratio (OR) with 95% confidence interval (CI) and corresponding *P*-value. Multivariable logistic regression models were corrected for tumor grade. All analyses were assessed at three thresholds for neurocognitive deficits: at ≤-1.0 standard deviation (SD), ≤-1.5 SD and ≤-2.0 SD.

**Supplementary table 12 Univariable and multivariable analyses correcting for tumor grade in IDH-wild type patients**

|  | **Univariable analyses** |  | **Multivariable analyses** | |
| --- | --- | --- | --- | --- |
| **Domain (≤-1.0 SD)** | **OR (95% CI)** | ***P*-value** | **OR (95% CI)** | ***P*-value** |
| Any domain  Attention and executive functioning  Language  Memory  Psychomotor speed  Visuospatial functioning | 1.455 (0.140 – 15.109)  0.230 (0.060 – 0.890)  1.210 (0.0364 – 4.020)  0.579 (0.153 – 2.191)  0.840 (0.263 – 2.679)  1.050 (0.304 – 3.622) | 0.754  0.033*  0.756  0.421  0.768  0.938 | 2.063 (0.146 – 29.180)  0.231 (0.059 – 0.907)  1.171 (0.331 – 4.145)  0.560 (0.116 – 2.694)  0.879 (0.258 – 2.996)  1.238 (0.308 – 4.980) | 0.592  0.036*  0.807  0.469  0.836  0.764 |
| **Domain (≤-1.5 SD)** | **OR (95% CI)** | ***P*-value** | **OR (95% CI)** | ***P*-value** |
| Any domain  Attention and executive functioning  Language  Memory  Psychomotor speed  Visuospatial functioning | 0.933 (0.203 – 4.285)  0.563 (0.173 – 1.827)  0.638 (0.188 – 2.161)  0.445 (0.137 – 1.443)  0.831 (0.257 – 2.685)  1.250 (0.368 – 4.246) | 0.929  0.338  0.470  0.178  0.757  0.721 | 1.104 (0.184 – 6.616)  0.568 (0.164 – 1.969)  0.595 (0.169 – 2.095)  0.433 (0.124 – 1.512)  0.865 (0.255 – 2.930)  1.393 (0.385 – 5.044) | 0.914  0.372  0.419  0.189  0.816  0.614 |
| **Domain (≤-2.0 SD)** | **OR (95% CI)** | ***P*-value** | **OR (95% CI)** | ***P*-value** |
| Any domain  Attention and executive functioning  Language  Memory  Psychomotor speed  Visuospatial functioning | 0.443 (0.122 – 1.612)  0.521 (0.152 – 1.787)  0.381 (0.072 – 2.020)  0.268 (0.065 – 1.097)  0.727 (0.219 – 2.412)  2.431 (0.684 – 8.643) | 0.216  0.300  0.257  0.067**  0.603  0.170 | 0.395 (0.088 – 1.772)  0.521 (0.142 – 1.912)  0.352 (0.065 – 1.915)  0.267 (0.064 – 1.116)  0.750 (0.209– 2.691)  2.726 (0.729 – 10.193) | 0.225  0.326  0.227  0.070**  0.659  0.136 |

Results of univariable and multivariable analyses of neuroligin-3 for deficits in any domain and all domains in the isocitrate dehydrogenase-wild type subgroup are reported separately as odds ratio (OR) with 95% confidence interval (CI) and corresponding *P*-value. Multivariable logistic regression models were corrected for tumor grade. All analyses were assessed at three thresholds for neurocognitive deficits: at ≤-1.0 standard deviation (SD), ≤-1.5 SD and ≤-2.0 SD. Significant *P*-values are marked with an asterisk(*). Near significant results (*P*<0.10) are marked with double asterisks (**).

**Supplementary table 13 Multivariable analyses correcting for tumor grade, volume and location in IDH-wild type patients – memory**

| Determinant | OR (95% CI) | *P*-value |
| --- | --- | --- |
| Neuroligin-3 | 0.105 (0.016 – 0.704) | 0.020* |
| WHO 2016 Grade 4 glioblastoma IDH-WT (vs Grade 2/3 astrocytoma IDH-WT) | 3.564 (0.314 – 40.505) | 0.306 |
| Tumor volume (cm^3^) | 1.00 (1.00 – 1.00) | 0.138 |
| Location Right hemisphere (vs left) | 2.833 (0.560 – 14.340) | 0.208 |

Results of multivariable analysis of neuroligin-3 for memory deficits at ≤-2.0 standard deviation in the isocitrate dehydrogenase-wild type (IDH-WT) subgroup are reported as odds ratio (OR) with 95% confidence interval (CI) and corresponding *P*-value. Multivariable logistic regression models were corrected for tumor grade, tumor volume and location on T_2_-weighted Fluid-Attenuated Inversion Recovery scans. Significant *P*-values are marked with an asterisk(*). WHO = World Health Organization; Gr = Grade; GBM = glioblastoma; A = astrocytoma.

**Supplementary table 14 Multivariable analyses correcting for tumor grade, volume and location in IDH-wild type patients – attention and executive functioning**

| Determinant | OR (95% CI) | *P*-value |
| --- | --- | --- |
| Neuroligin-3 | 0.231 (0.055 – 0.965) | 0.045* |
| WHO 2016 Grade 4 glioblastoma IDH-WT (vs Grade 2/3 astrocytoma IDH-WT) | 1.579 (0.234 – 10.663) | 0.639 |
| Tumor volume (cm^3^) | 1.00 (1.00 – 1.00) | 0.575 |
| Location Right hemisphere (vs left) | 0.669 (0.148 – 3.011) | 0.600 |

Results of multivariable analysis of neuroligin-3 for attention and executive functioning deficits at ≤-1.0 standard deviation in the isocitrate dehydrogenase-wild type (IDH-WT) subgroup are reported as odds ratio (OR) with 95% confidence interval (CI) and corresponding *P*-value. Multivariable logistic regression models were corrected for tumor grade, tumor volume and location on T_2_-weighted Fluid-Attenuated Inversion Recovery scans. Significant *P*-values are marked with an asterisk(*). WHO = World Health Organization; Gr = Grade; GBM = glioblastoma; A = astrocytoma.

**Supplementary table 15 Multivariable analyses correcting for tumor grade, volume and location, and presence of epilepsy in IDH-wild type patients – memory**

| Determinant | OR (95% CI) | *P*-value |
| --- | --- | --- |
| Neuroligin-3 | 0.072 (0.007 – 0.729) | 0.026* |
| WHO 2016 Grade 4 glioblastoma IDH-WT (vs Grade 2/3 astrocytoma IDH-WT) | 2.461 (0.201 – 30.183) | 0.481 |
| Tumor volume (cm^3^) | 1.000 (1.000 – 1.000) | 0.242 |
| Location Right hemisphere (vs left) | 2.508 (0.440 – 14.311) | 0.301 |
| Presence of epilepsy (at presentation) | 0.495 (0.106 – 2.303) | 0.370 |

Results of multivariable analysis of neuroligin-3 for memory deficits at ≤-2.0 standard deviation in the isocitrate dehydrogenase-wild type (IDH-WT) subgroup are reported as odds ratio (OR) with 95% confidence interval (CI) and corresponding *P*-value. Multivariable logistic regression models were corrected for tumor grade, tumor volume and location on T_2_-weighted Fluid-Attenuated Inversion Recovery scans, and the presence of epilepsy at presentation. Significant *P*-values are marked with an asterisk(*). WHO = World Health Organization.

**Supplementary table 16 Multivariable analyses correcting for tumor grade, volume and location, and presence of epilepsy in IDH-wild type patients – attention and executive functioning**

| Determinant | OR (95% CI) | *P*-value |
| --- | --- | --- |
| Neuroligin-3 | 3.790 (0.775 – 18.540) | 0.100 |
| WHO 2016 Grade 4 glioblastoma IDH-WT (vs Grade 2/3 astrocytoma IDH-WT) | 0.455 (0.062 – 3.332) | 0.438 |
| Tumor volume (cm^3^) | 1.000 (1.000 – 1.000) | 0.207 |
| Location Right hemisphere (vs left) | 0.793 (0.137 – 4.582) | 0.795 |
| Presence of epilepsy (at presentation) | 0.477 (0.060 – 3.769) | 0.482 |

Results of multivariable analysis of neuroligin-3 for attention and executive functioning deficits at ≤-1.0 standard deviation in the isocitrate dehydrogenase-wild type (IDH-WT) subgroup are reported as odds ratio (OR) with 95% confidence interval (CI) and corresponding *P*-value. Multivariable logistic regression models were corrected for tumor grade, tumor volume and location on T_2_-weighted Fluid-Attenuated Inversion Recovery scans, and the presence of epilepsy at presentation. WHO = World Health Organization.

**Supplementary table 17 Multivariable analyses correcting for tumor grade, volume and location, and an interaction term between neuroligin-3 and presence of epilepsy in IDH-wild type patients – memory**

| Determinant | OR (95% CI) | *P*-value |
| --- | --- | --- |
| Neuroligin-3 | 0.157 (0.011 – 2.273) | 0.174 |
| WHO 2016 Grade 4 glioblastoma IDH-WT (vs Grade 2/3 astrocytoma IDH-WT) | 2.595 (0.203 – 33.232) | 0.464 |
| Tumor volume (cm^3^) | 1.000 (1.000 – 1.000) | 0.306 |
| Location Right hemisphere (vs left) | 2.499 (0.432 – 14.469) | 0.307 |
| Presence of epilepsy (at presentation) | 0.593 (0.122 – 2.880) | 0.517 |
| Presence of epilepsy*Neuroligin-3 | 0.00 (0.00 – ∞) | 0.999 |

Results of multivariable analysis of neuroligin-3 for memory deficits at ≤-2.0 standard deviation in the isocitrate dehydrogenase-wild type (IDH-WT) subgroup are reported as odds ratio (OR) with 95% confidence interval (CI) and corresponding *P*-value. Multivariable logistic regression models were corrected for tumor grade, tumor volume and location on T_2_-weighted Fluid-Attenuated Inversion Recovery scans, and epilepsy at presentation, including an interaction term with neuroligin-3 (Epilepsy*Neuroligin-3). WHO = World Health Organization.
